# Transcriptomic responses of the liver and adipose tissues to altered carbohydrate-fat ratio in diet: an isoenergetic study in young rats

**DOI:** 10.1186/s12263-017-0558-2

**Published:** 2017-04-08

**Authors:** Mitsuru Tanaka, Akihito Yasuoka, Manae Shimizu, Yoshikazu Saito, Kei Kumakura, Tomiko Asakura, Toshitada Nagai

**Affiliations:** 1 Nissin Global Innovation Center, Nissin Foods Holdings, 2100 Tobukimachi, Hachioji-shi, Tokyo 192-0001 Japan; 2 Project on Health and Anti-Aging, Kanagawa Academy of Science and Technology, Life Science and Environment Research Center (LiSE) 4F C-4, 3-25-13 Tonomachi, Kawasaki-ku, Kawasaki, Kanagawa 210-0821 Japan; 3 Department of Health and Nutrition, Takasaki University of Health and Welfare, 37-1 Nakaorui-machi, Takasaki, Gunma 370-0033 Japan; 4 Department of Applied Biological Chemistry, Graduate School of Agricultural and Life Sciences, The University of Tokyo, 1-1-1 Yayoi, Bunkyo-ku, Tokyo 113-8657 Japan

**Keywords:** Transcriptome, Carbohydrate-fat ratio, Liver, White adipose tissue, Brown adipose tissue

## Abstract

**Background:**

To elucidate the effects of altered dietary carbohydrate and fat balance on liver and adipose tissue transcriptomes, 3-week-old rats were fed three kinds of diets: low-, moderate-, and high-fat diets (L, M, and H) containing a different ratio of carbohydrate-fat (C-F) (65:15, 60:20, and 35:45 in energy percent, respectively).

**Methods:**

The rats consumed the diets for 9 weeks and were subjected to biochemical and DNA microarray analyses.

**Results:**

The rats in the H-group exhibited lower serum triacylglycerol (TG) levels but higher liver TG and cholesterol content than rats in the L-group. The analysis of differentially expressed genes (DEGs) between each group (L vs M, M vs H, and L vs H) in the liver revealed about 35% of L vs H DEGs that were regulated in the same way as M vs H DEGs, and most of the others were L- vs H-specific. Gene ontology analysis of these L vs H DEGs indicated that those related to fatty acid synthesis and circadian rhythm were enriched. Interestingly, about 30% of L vs M DEGs were regulated in a reverse way compared with L vs H and M vs H DEGs. These reversed liver DEGs included M-up/H-down genes (*Sds* for gluconeogenesis from amino acids) and M-down/H-up genes (*Gpd2* for gluconeogenesis from glycerol, *Agpat9* for TG synthesis, and *Acot1* for beta-oxidation). We also analyzed L vs H DEGs in white (WAT) and brown (BAT) adipose tissues and found that both oxidation and synthesis of fatty acids were inhibited in these tissues.

**Conclusions:**

These results indicate that the alteration of dietary C-F balance differentially affects the transcriptomes of metabolizing and energy-storing tissues.

**Electronic supplementary material:**

The online version of this article (doi:10.1186/s12263-017-0558-2) contains supplementary material, which is available to authorized users.

## Background

Availability of body carbohydrate (C) and fat (F) for energy production varies depending on the animal’s circumstances. Fat is mainly consumed during resting conditions at about 90% of total energy; however, this ratio can be rapidly decreased to nearly 10% through acute bouts of exercise and substituted by the energy supply from aerobic or anaerobic respiration of C [7, 38]. Under fasting conditions, carbohydrate is depleted within a day, and about four fifths of basal metabolic rate is maintained by fat and the rest by amino acids for several days [4]. These metabolic switches of energy source between C and F are more interchangeable than protein (P) or amino acids because of the metabolic linkage mediated by the key organic substances: glycerol-3-phosphate both as the product of triacylglycerol (TG) hydrolysis and as the substrate for gluconeogenesis, NADP(H) both as the hydrogen acceptor of the pentose phosphate pathway and as the hydrogen donor for fatty acid (FA) synthesis, and acetyl-CoA as the activated substrate of the TCA cycle and of FA synthesis. Thus, dietary C to F ratio (C-F ratio) has a considerable effect on the energy homeostasis of animals.

Generally, experimental rodents accept diets composed of energetic C-F ranging from 50:30 to 70:10 to provide a constant energy ratio of 20% P [39]. In rodents, AIN93G (C:F:P = 64:16:20) during rapid growth, pregnancy, and lactation and AIN93M (C:F:P = 76:9:15) during maintenance were often used for standard diets [28]. Keeping this P energy ratio over 15% is critical for normal growth of adolescent animals [13, 23, 29]. But effects of an altered C-F on metabolic parameters differ depending on dietary fat species such as soybean and corn oils of plant origin, and beef tallow and lard of animal origin. It was shown that a high-fat diet (HFD, C:F:P = 30:40:20) made of lard was more deleterious to insulin resistance and hepatic steatosis than an HFD made of soybean oil in comparison with a low-fat diet (LFD, C:F:P = 14:64:22) [45, 50]. Deol et al. reported that an HFD (C:F:P = 43:40:16) containing soybean oil and hydrogenated coconut oil at 1:1 ratio was more obesogenic than an HFD mainly containing hydrogenated coconut oil [10]. These differences were considered to be caused by the lipid composition of the dietary fat [1, 8, 12, 17, 32, 34]. Polyunsaturated FAs (PUFAs) are the main contributors to the physiological activity of dietary fat; soybean oil contains 15% saturated FAs and 55% PUFAs, while lard contains 40% saturated FAs and 10% PUFAs. Duivenvoorde et al. showed that an HFD with predominantly saturated FAs increased ectopic fat storage, liver damage, and adipocyte size as compared to an HFD with predominantly PUFAs and reduced response flexibility to fast re-feeding and oxygen restriction [11]. Especially, eicosapentaenoic (EPA) and docosahexaenoic acid (DHA) were reported to reduce insulin resistance and hepatic steatosis [26, 31]. Though small in percentage, sterols are critical factors for animal lipid homeostasis; the soybean oil used in our study contained 0.0024% cholesterol and 0.33% phytosterols, while the lard contained 0.086% cholesterol and no phytosterols. Specifically, phytosterols have been shown to exert beneficial effects on lipid homeostasis under metabolically stressed conditions such as an HFD containing predominantly saturated FAs [5, 6, 16, 27, 36]. However, there are few studies on the transcriptomic effects of a gradual change in the C-F under more moderate conditions, such as the use of diets containing natural plant oils or restricted feeding [30, 37]. In the present study, we conducted an isoenergetic study using a soybean oil-rich diet and found fewer deleterious effects on tissue metabolism but a drastic change in the tissue transcriptome.

## Methods

### Animals

Three-week-old male Wistar rats (Charles River Laboratories Japan, Kanagawa, Japan) were housed in a temperature- and humidity-controlled room with a 12-h light-dark cycle (light 06:30–18:30, dark 18:30–06:30). All animal experimental protocols were approved by the Animal Use Committee of the Takasaki University of Health and Welfare.

### Experimental procedure

The rats were acclimated to the laboratory environment for a week with chow diets (MF, Oriental yeast, Tokyo, Japan). The animals were divided into three groups so that the average body weights of each group were equal to each other before being given diets with different C-F energy ratios: low (L) 65:15, moderate (M) 60:20, and high (H) 35:45 fat diet groups. The rats were fed diets ad libitum for a week. Then, the L-group was fed ad libitum and the other groups were fed isoenergetically compared with the L-group for 9 weeks. The diets were purchased from Research Diets, Inc. (New Brunswick, NJ, USA). Detailed compositions of each diet are shown in Additional file 1. Diets were removed 17 h before dissection, and the rats were sacrificed to collect the blood, liver, white adipose tissue (WAT), and brown adipose tissue (BAT). Because an obviously decreased dietary intake was observed for two rats belonging to the M- or H-groups (M_7 and H_11 in identical number), the use of these two rats were not included in all analyses to achieve consistency in the isoenergetic study (*n* = 4–5 in each group). Serum and plasma were extracted using standard methods and separated from whole blood. Small hepatic pieces were immersed into RNAlater (Qiagen, Tokyo, Japan). The rest hepatic pieces, WAT, and BAT were frozen immediately after extirpation using liquid nitrogen. All samples were stored at −80 or −150 °C until analysis.

### Measurement of blood biochemical parameters

All blood biochemical parameters, except insulin, listed in Table 1, were analyzed by Nagahama Life Science (Shiga, Japan). Plasma was used to measure glucose, pyruvic acid, total lipids, phospholipids, and total ketone bodies. Other parameters were assayed using the serum. Serum insulin levels were measured by using the rat insulin ELISA kit (Morinaga Institute of Biological Science, Kanagawa, Japan).

**Table 1:**
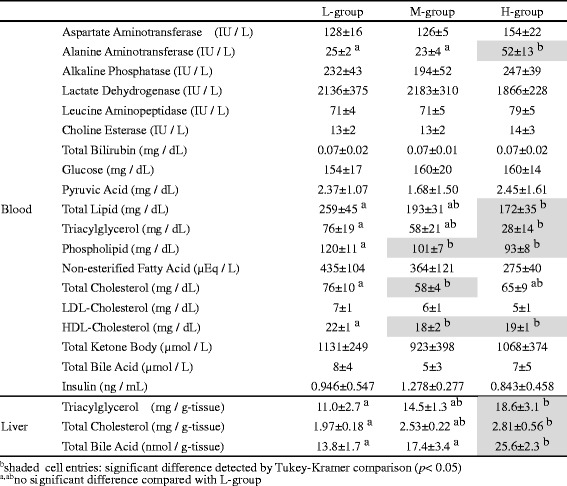
Blood and liver biochemical analysis

### Measurement of hepatic lipids

Hepatic lipids were extracted according to a previous method [14]. Briefly, 100 mg of frozen hepatic pieces were homogenized in 2 mL of cooled chloroform-methanol solution (2:1) using a multibead shocker (Yasui Kikai Corporation, Osaka, Japan). Filtered samples were adjusted to 4 mL with chloroform-methanol solution and were washed with 0.8 mL of purified water. Subsequent washes were performed by adding 3.75 mL of chloroform-methanol-water solution (2:1:0.75), and the resulting extracts were dried by evaporation. Extracted lipids were resolved with 1 mL of isopropanol.

Hepatic TG, total cholesterol, and total bile acids were measured using Cholestest TG, Cholestest CHO (Sekisui Medical, Tokyo, Japan), and total bile acids assay kits (Diazyme Laboratories, Poway, CA, USA), respectively.

### DNA microarray assay

Total RNA was isolated from each immersed hepatic piece, WAT, and BAT by TRIzol reagent (Invitrogen Japan, Tokyo, Japan) and purified using RNeasy mini kits (Qiagen). Anti-sense RNA was synthesized from 100 or 200 ng of purified total RNA, and biotinylated complementary RNA (cRNA) was obtained using a GeneChip 3’IVT Express Kit (Affymetrix, Santa Clara, CA, USA). The cRNA was fragmented and hybridized to a GeneChip Rat Genome 230 2.0 Array (Affymetrix) for 16 h at 45 °C. The arrays were washed and stained with phycoerythrin using the GeneChip Fluidics Station 450 (Affymetrix) and submitted to scanning on an Affymetrix GeneChip Scanner 3000 7G. The Affymetrix GeneChip Command Console Software was used to make CEL files.

### DNA microarray data analysis

The CEL files derived from the liver, WAT, and BAT were quantified using robust multi-array average (RMA), factor analysis for robust microarray summarization (quantile normalization, qFARMS), and GCRMA, respectively [19, 22, 46], using the statistical language R (2.7.1) (http://www.r-project.org/) (R [35]), and Bioconductor (2.2) (http://www.bioconductor.org/) [15]. Hierarchical clustering was performed using the pvclust function in R [41]. The rank products (RP) method was used to identify differentially expressed gene probe sets of the quantified data [3]. The probe sets with a false discovery rate (FDR) <0.05 were considered to be differentially expressed between each group (L vs M, M vs H, and L vs H).

The up- and downregulated probe sets picked out at FDR < 0.05 were functionally classified by the Biological Process in Gene Ontology (GO) with the Functional Annotation Tool of the Database for Annotation, Visualization, and Integrated Discovery (DAVID) [9, 21] and Quick GO (http://www.ebi.ac.uk/QuickGO/) [20]. In analysis of the liver, EASE scores, which are modified Fisher’s exact test *p* values were used to extract statistically overrepresented GO terms, and GO terms with *p* values <0.01 were regarded as significantly enriched. In analysis of WAT and BAT, Benjamini-Hochberg correction *p* values were used to extract statistically overrepresented GO terms, and GO terms with *p* values <0.05 were regarded as significantly enriched.

Predicted upstream regulators among liver and adipose tissue transcriptomes were analyzed using Qiagen’s Ingenuity Pathway Analysis (IPA, Qiagen, https://www.qiagenbioinformatics.com/products/ingenuity-pathway-analysis/). Activation *z*-scores were calculated as a measure of upstream regulators analysis. An absolute z-score ≥2.5 was judged as significantly activated or inhibited. Common upstream regulators that were predicted to be activated or inhibited in the liver, WAT, and BAT were picked out from a list of all upstream regulators.

### Statistical analysis

The results are shown as the means ± SDs. One-way ANOVA was used to assess the differences among three groups, and Tukey-Kramer comparison was used for pairwise comparisons between multiple groups. Differences at *p* ≤ 0.05 were considered to be significant.

## Results

### Characterization of hepatic genes affected by the altered balance of carbohydrate and fat in the diet

Rats were fed three kinds of diets containing different ratios of C-F in constant total energy (L, M, and H, Additional file 1). In our preliminary experiment of feeding ad libitum, energy intakes (Kcal/g-BW) were almost the same among the three groups from week 2 to week 4. Therefore, rats were pair-fed to keep by isoenergetic conditions, and dietary restriction derived from pair-feeding has not been occurred. During the experimental period of 9 weeks, the rats in each group showed no between-group differences in body weight (Additional file 2a, b). Also, the liver and the WAT weights showed no differences among groups (Additional file 2b). Biochemical analysis of the blood revealed differences in several markers among experimental groups (Table 1). The H-group showed higher levels of alanine aminotransferase (ALT) and lower levels of TG, phospholipid, and HDL cholesterol (HDL-Chl). The M-group showed lower levels of phospholipids, total Chl, and HDL-Chl. In addition, the liver biochemical analysis indicated increases in TG, total Chl, and total bile acid (BA) in the H-group. Serum insulin levels did not change among the three groups (Table 1).

**Fig. 1:**
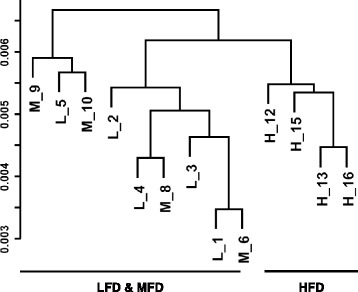
Cluster analysis of each liver transcriptome in experimental groups. RMA-normalized expression data were subjected to hierarchical clustering analysis and represented in a dendrogram. Each sample name consists of a letter corresponding to the feeding condition (L, LFD; M, MFD; H, HFD) and a number corresponding to the individual rat. The vertical scale represents the distance between each transcriptome

**Fig. 2:**
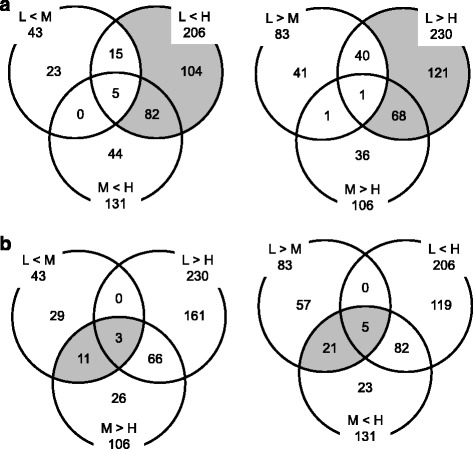
Number of liver probe sets that were differentially expressed between experimental groups. **a** Coincidence of DEGs among experimental groups. The subsets of DEGs specific to the L vs H change are indicated by *shaded areas*. **b** Oppositely regulated DEGs (*shaded areas*)

The liver transcriptomes of the H-group were segregated from those in the L- and M-groups in the cluster dendrogram (Fig. 1). To dissect this overall difference in transcriptomes at a single gene level, we analyzed the coincidence of differentially expressed genes (DEGs) estimated from the comparison among L-, M-, and H-groups (Fig. 2a). The DEGs were termed according to the experimental groups and the number of members. For example, LM43 + 83 formed the smallest population among MH131 + 106 and LH206 + 230, and shared about half of the members (15 + 5 and 40 + 1) with LH206 + 230. In contrast, about one third of LH206 + 230 members were included by MH131 + 106. This indicates that the transcriptomic change from L to H is more similar to the change from M to H than the change from L to M.

Then, we examined the function of the DEGs specific to the L vs H change (LH186 + 189 probe sets, Fig. 2a shaded area) using GO enrichment analysis [9, 21]. As a result, 53 genes were attributed to the nine GO terms located at the lowest position in the hierarchy (Table 2). Among these GO terms, four terms were related to lipid metabolism (GO0019216, 0006633, 0008203, and 0033189). The enriched genes included 5 + 3 metabolic enzyme genes. *Fads1*, *Msmo1*, *Cyp7b1*, *Idi1*, and *Sqle* were upregulated and *Cyp4a1*, *Elovl5*, and *Scd1* were downregulated in the H-group (Additional file 3, shaded cell entries), suggesting down- or upregulation of PUFA synthesis and upregulation of Chl/BA synthesis. In addition, *Apoa4*, a key regulator of enteric and hepatic TG transportation was downregulated in the H-group. Other members of this category were mostly regulatory protein genes such as *Prkaa1* (protein kinase, AMP-activated, alpha 1) and *2*, *Srebf1* (sterol regulatory element-binding transcription factor 1), *Il1a* (interleukin-1 alpha), glucocorticoid receptor, *Lepr* (leptin receptor), and *Dusp1* (MAPK phosphatase); among these, only Srebf1 was upregulated and the others were downregulated in the H-group. There were 6 genes that belong to the GO term, circadian rhythm (GO0007623). Upregulation of *Arntl/Clock*, *Npas2*/Clock paralog, and *Egfr* (epidermal growth factor receptor) as day genes and downregulation of *Prf1*(perforin 1), *Per* (period circadian clock) *1* and *2* as night genes in the H-group was consistent with the reversed expression pattern of these genes at the time point of tissue sampling (zeitgeber time 3) [2]. Fourteen genes were identified as those related to RNA polymerase II-dependent transcription (GO0045944 and 0000122); among these, only *Ppargc1b* (*Pgc1b*) was upregulated, and the others were downregulated in the H-group.

**Table 2:**
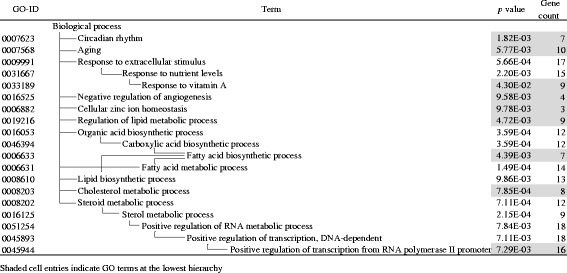
Significantly enriched GO terms found in liver LH186 + 189 genes

Besides the significant enrichment of LH186 + 189 genes to the GO terms related to lipid metabolism, LM43 + 83 genes were hard to analyze in this way because of the small population. We then dissected these genes with reference to the regulation of M vs H or L vs H DEGs (Fig. 2b). It was revealed that 14 + 26 probe sets were reversely regulated compared with L vs H or M vs H DEGs (Table 3). These sets included 11 metabolic enzyme genes (shaded cell entries): *Sds* (serine dehydratase) for utilization of glycogenic amino acids; *Acot1* (acyl-CoA thioesterase 1) for negative regulation of beta-oxidation; *Acsm2* (acyl-CoA synthetase medium-chain family member 2) for positive regulation of FA synthesis; *Agpat9* (1-acylglycerol-3-phosphate *O*-acyltransferase 9) for TG synthesis; *Gpd2* (glycerol-3-phosphate dehydrogenase 2, mitochondrial) for gluconeogenesis from glycerol; and *Cyp2b1*, *Akr7a3*, *Cyp26b1*, *Cyp4a8*, *Gstt3*, and *Sqrdl* for detoxication. The other genes were involved in more diversified functions. This result indicates that the M-group is situated in a nutritional condition that controls the regulatory switching of these metabolic genes.

**Table 3:**
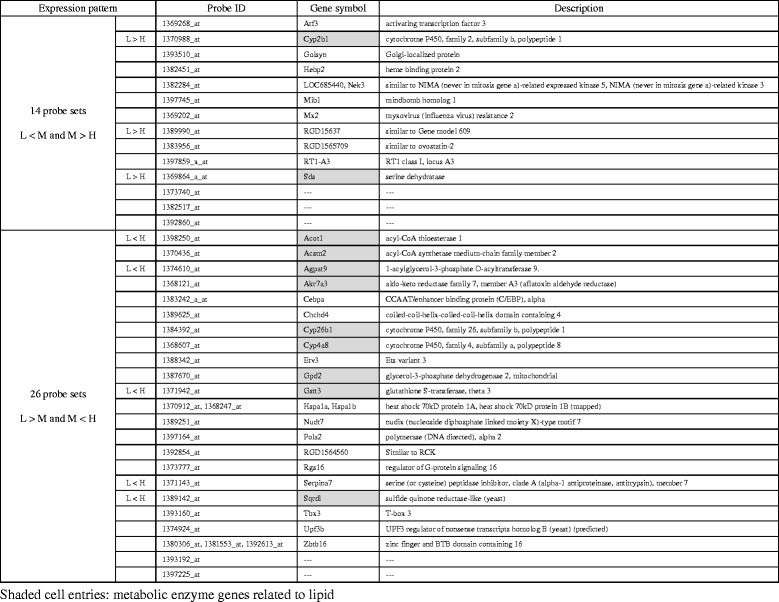
The list of the reversely regulated liver LM43 + 83 genes

### Response of the adipose tissue transcriptomes to the increased ratio of fat to carbohydrate

Because the hepatic transcriptome response as described above suggested some change in energetic interaction with other tissues such as adipose tissues, we analyzed the transcriptomes of WAT and BAT in each experimental condition (Table 4). The L vs H DEGs of these tissues were subjected to GO term enrichment analysis as in the case of the liver. WAT LH235 + 336 DEGs showed marked enrichment to the terms related to lipid metabolism (42 genes to GO0008610, 0006635, and 0045444) (Table 5), and most of the metabolic enzyme genes were downregulated in the H-group (Additional file 4). It is possible that both lipid synthesis and beta-oxidation were suppressed in this condition. Other characteristics of WAT LH235 + 336 DEGs were the high frequency of regulatory protein genes in the GO terms related to glucose metabolism (GO006006) (*Pik3r1*, *Lep*, *Il6st*, *Igf2*, *Atf3*, *Crem*, *Pdk1*, and *Ppp1r1a*, totally 8 genes/another 13 genes), and insulin signaling (GO0032868) (*Lyn*, *Foxo1*, *Acvr1c*, *Pde3b*, and *Shc1*, totally 5 genes/another 9 genes). Most of these genes were downregulated in the H-group except *Lep* encoding satiety hormone leptin, *Il6st* encoding IL-6 inflammatory signal transducer, and *Lyn* encoding tyrosine kinase. There were 12 genes attributed to the GO terms related to bone formation (GO0060348 and GO0001503).

**Table 4:** Differentially expressed genes in the liver and in the adipose tissues

Tissue	L < H	L > H
Liver	206	230
WAT	235	336
BAT	212	405

**Table 5:**
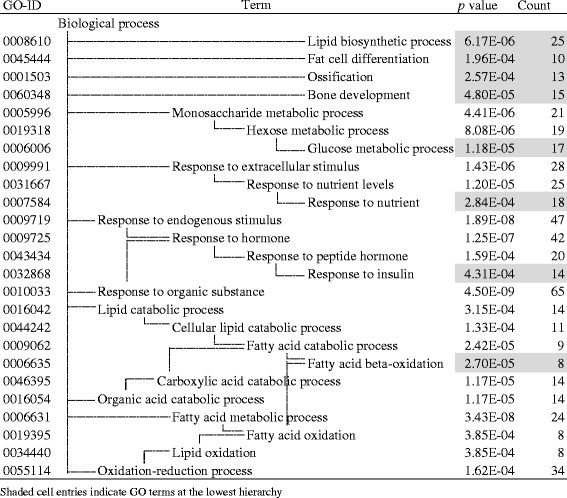
Significantly enriched GO terms found in WAT LH235 + 336 genes

BAT LH212 + 405 DEGs exhibited a regulatory pattern similar to that of WAT DEGs (Table 6), where all of the enzyme genes related to lipid metabolism were downregulated in the H-group (24 genes in GO0006631 and 0006695, shaded cell entries in Additional file 5). The other 23 enzyme genes were in the oxidation-reduction category (GO0055114) of which 15 genes were downregulated in the H-group. BAT DEGs also contained another 46 genes classified as organic substance responsive components (GO0010033) that encode regulatory proteins, transcription factors (SREBF2, glucagon receptor), and transporters. The remainder was 12 genes for muscle contraction (GO0006936) such as *actin*, *myosin*, and *troponin* genes.

**Table 6:**
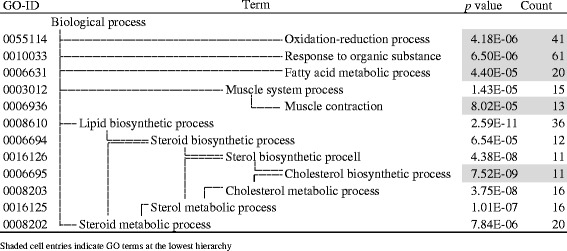
Significantly enriched GO terms found in BAT LH212 + 405 genes

### Search for upstream regulators common among the liver and adipose tissues

Given the results of GO analysis that the H-diet generally induced the upregulation of FA unsaturation and Chl synthesis in the liver (Additional file 3) and the downregulation of FA synthesis in the adipose tissues (Additional files 4 and 5), we assessed whether these gene regulations were caused by some biological signals common among these tissues using the Ingenuity Pathway Analysis (IPA). Table 7 lists the IPA upstream regulators that were predicted to be activated or repressed (absolute z-score > 2.5) from the input of L vs H DEGs (Table 4). Relatively high *z*-scores were observed with LY294002 (PI3 kinase inhibitor) in WAT (3.07) and BAT (2.73) [44], suggesting the inhibition of insulin signaling in the H-group. This is consistent with the result that two well-known components of insulin downstream signaling (SREBF1 for FA synthesis and SREBF2 for Chl synthesis) were inactivated (negative *z*-scores) both in WAT (−3.68 and −4.18) and BAT (−3.52 and −4.17). It is also notable that INSIG (insulin-induced gene protein) 1 and 2, which play roles as repressors of SREBF [48, 49], seemed to be activated in BAT (3.61 and 2.93). In addition, pirinixic acid, a specific agonist of PPAR (peroxisome proliferator-activated receptor) alpha, was detected as a WAT/BAT common upstream regulator. The negative *z*-scores for pirinixic acid (−3.07 in WAT and −2.99 in BAT) suggest the repression of this process. The liver transcriptome showed relatively low absolute *z*-scores except for peptidylprolyl isomerase F (PPIF or cyclophilin D) (*z*-score = 2.83).

**Table 7:**
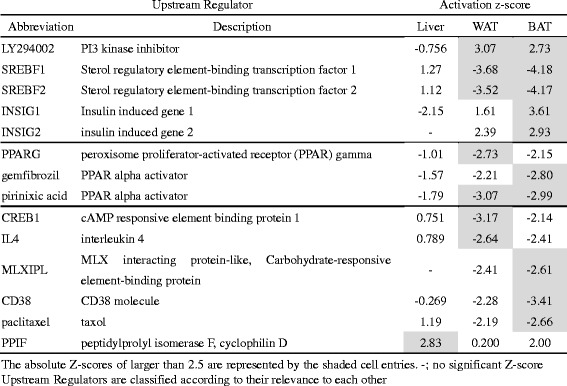
Comparison of IPA upstream regulators among the liver and the adipose tissue transcriptomes

## Discussion

We have analyzed the transcriptomic responses of the liver and adipose tissues to an increased ratio of F to C under isoenergetic conditions. In this study, three types of diets were adjusted with soybean oil to construct the C-F ratios, since it is the major oil in human diets. Soybean oil has some beneficial effects [45, 50], and hepatic transcriptomes can be influenced by oil and fat profiles [18]. Although the fatty acid profile was different among three diets because of identical quantities of lard rich in saturated FA, it is crucial that the main energy resource was changed from C to F. The rats showed no between-group differences in body weight or in relative tissue weight (Additional file 2b); however, higher serum ALT levels were observed in the H-group compared with the L- and M-groups (Table 1). Because no significant fluctuations were observed among the other damage markers, the liver damage in the H-group seems to be limited in extent. This is in accordance with the fact that no significant enrichment of DEGs detected in GO terms related to liver damage, such as inflammation or fibrosis [25].

**Fig. 3:**
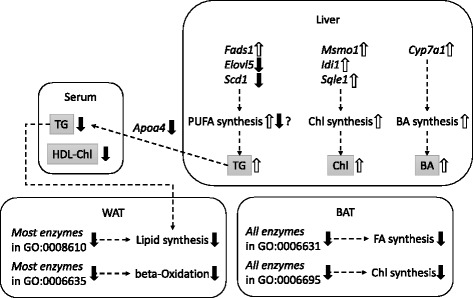
Transcriptomic and metabolic changes in H-condition compared to L-condition. *Shaded molecules* indicate the metabolites, and others indicate the transcripts specific to L vs H change (liver LH186 + 189, WAT LH235 + 336, and BAT LH212 + 405). *Upward arrows* indicate the H-up genes (*italics*) or predicted pathways compared to L-condition, and vice versa. *TG* triacylglycerol, *Chl* cholesterol, *BA* bile acid, *FA* fatty acid, *PUFA* polyunsaturated fatty acid

Interestingly, H-group rats exhibited a significant biochemical characteristic relevant to lipid homeostasis: lower TG and HDL-Chl levels in the sera and higher TG, total Chl, and total BA content in the liver than in the L-group (Table 1). Our transcriptomic analysis suggested the upregulation of Chl/BA synthesis in the liver (Table 2 and Additional file 3), the downregulation of lipid synthesis and beta-oxidation in WAT (Table 5 and Additional file 4), and the downregulation of Chl biosynthesis in BAT (Table 6 and Additional file 5). The former liver transcriptomic response may facilitate acetyl-CoA consumption via Chl synthesis and BA secretion (Fig. 3) [43]. Moreover, the downregulation of *Scd1* and *Elovl5* indicates suppression of de novo synthesis and elongation of monounsaturated FAs, while the upregulation of *Fads1* implies facilitation of C20 PUFAs (precursors of bioactive eicosanoids) synthesis from 18:2 n-6 linoleic acid, rich in H-diet, with the help of *Fads2* [24]. These results suggest that the hepatic transcriptome was regulated not only by the C-F ratios but also by the fatty acid profiles of the diets. The downregulation of *Apoa4* may inhibit export of TG from the liver leading to the decrease in serum TG level and the increase in liver TG content (Fig. 3) [42]. The latter responses of adipose tissues may suppress FA release to the sera.

A comparison of L vs M transcriptomes in liver showed 126 (43 + 83) genes as differentially expressed (Fig. 2); this was less than the number of differentially expressed genes as compared to M vs H (131 + 106 genes) and L vs H (206 + 230 genes). This means that the transcriptome of the L-group was more closely related to that of the M-group than H-group (Fig. 1). Then, we analyzed LM43 + 83 DEGs to clarify C-F ratio dependency of hepatic transcriptome and we found 32 reversely regulated genes (i.e., upregulated in M-condition and downregulated in H-condition, or vice versa) (listed in Table 3). These reversely regulated liver DEGs can exert potential effects on lipid homeostasis; the upregulation of *Acot1*, *Acsm2*, and *Agpat9* in the H-group may increase TG accumulation in the liver. Also, the role of LM43 + 83 DEGs in macronutrient conversion (e.g., amino acid to C and F to C) should be emphasized because our study was conducted under the isoenergetic conditions. In this context, the downregulation of *Sds* in the H-group may reduce utilization of amino acids for gluconeogenesis, and the upregulation of *Gpd2* in the H-group may increase gluconeogenesis from glycerol produced by TG hydrolysis. Because the expression pattern of these genes was biphasic, the regulation of these metabolisms may have a balancing point close to the M-condition. As we used outbred Wistar rats, transcriptomic difference among the L-group and the M-group could be influenced by genetic or epigenetic differences between animals. Further indirect calorimetric studies with altered C-F ratios or animal strains are needed to clarify this metabolic regulation switching.

A question arising is whether these transcriptomic regulations are governed by any cellular signals common among these tissues. We computationally detected the downregulation of both insulin-PI3K-SREBF and PPAR alpha signals in the adipose tissues but not in the liver (Table 7). This suggests that both the anabolic signal of insulin (i.e., FA synthesis) and the catabolic signal of PPAR alpha (i.e., FA oxidation) are inhibited in adipose tissues. Because the rats in the H-group showed a growth rate (Additional file 2b) and serum insulin levels almost the same as in the L- and M-groups (Table 1), the suppression of insulin signals may be intrinsic to adipose tissues [33, 40, 47]. In the case of PPAR alpha signal, the low level of serum TG in the H-group might affect the concentration of FA in adipose tissues.

## Conclusions

To investigate the effects of altered dietary C-F ratio, we compared with L vs M and L vs H DEGs. We found that hepatic genes for gluconeogenesis and lipid metabolism were reversely regulated, indicating that a turning point for gene expression switching from C to F as energy source may exist in the M-condition (C:F = 60:20) or a C-F ratio around M.

L vs H analyses revealed that high-fat diet upregulated Chl/BA synthesis in the liver and downregulated lipid synthesis in WAT and BAT. Also, our computational search for upstream regulators in these tissues suggested that insulin and PPAR alpha signals were downregulated both in WAT and BAT in the H-group.

In conclusion, the liver and adipose tissues differentially adapts to altered C-F by changing their gene expressions and not by merely responding to endocrine signals.

## Additional files

Additional file 1: Composition of diets. (DOCX 17 kb)

Additional file 2: Physical parameters of the animals. a, Energy intake during the experimental period. The intakes of the rats in the M- and H-groups were restricted to the average intake of the rats in the L-group. Data for the M- and H-groups after day 0 were omitted. b, Body and tissue weights. The *inset* represents the relative tissue weights (percent to body weight) at the time of sacrifice (week 9). Values are represented as means ± SD (*n* = 4–5). (DOCX 89 kb)

Additional file 3: The list of liver LH186 + 189 genes that belongs to the GO terms located at the lowest level of hierarchy. (DOC 143 kb)

Additional file 4: The list of WAT LH235 + 336 genes that belong to the GO terms located at the lowest level of hierarchy. (DOC 190 kb)

Additional file 5: The list of BAT LH212 + 405 genes that belong to the GO terms located at the lowest level of hierarchy. (DOC 213 kb)

## Experimental conditions

C: Carbohydrate
F: Fat
P: Protein
LFD or L: Low-fat diet
MFD or M: Moderate-fat diet
HFD or H: High-fat diet

## Methods and biochemical terms

ALT: Alanine aminotransferase
BA: Bile acid
BAT: Brown adipose tissues
Chl: Cholesterol
DEGs: Differentially expressed genes
FA: Fatty acid
FDR: False discovery rate
GO: Gene ontology
HDL: High-density lipoprotein
INSIG: Insulin-induced gene protein
IPA: Ingenuity Pathway Analysis
PPAR: Peroxisome proliferator-activated receptor
PPIF: Peptidylprolyl isomerase F (cyclophilin D)
PUFA: Polyunsaturated fatty acid
SREBF: Sterol regulatory element-binding transcription factor
TG: Triacylglycerol
WAT: White adipose tissues

## Genes

*Acot1*: Acyl-CoA thioesterase 1
*Acsm2*: Acyl-CoA synthetase medium-chain family member 2
*Acvr1c*: Activin A receptor, type IC
*Agpat9*: 1-Acylglycerol-3-phosphate *O*-acyltransferase 9
*Akr7a3*: Aldo-keto reductase family 7, member A3 (aflatoxin aldehyde reductase)
*Apoa4*: Apolipoprotein A-IV
*Arntl/Clock*: Aryl hydrocarbon receptor nuclear translocator-like
*Atf3*: Activating transcription factor 3
*Crem*: cAMP responsive element modulator
*Cyp*: Cytochrome P450
*Dusp1*: Dual specificity phosphatase 1
*Egfr*: Epidermal growth factor receptor
*Elovl5*: ELOVL fatty acid elongase 5
*Fads1*: Fatty acid desaturase 1
*Foxo1*: Forkhead box O1A
*Gpd2*: Glycerol-3-phosphate dehydrogenase 2, mitochondrial
*Gstt3*: Glutathione S-transferase, theta 3
*Idi1*: Isopentenyl-diphosphate delta isomerase 1
*Igf2*: Insulin-like growth factor 2
*Il1a*: Interleukin 1 alpha
*Il6st*: Interleukin 6 signal transducer
*Lep*: Leptin
*Lepr*: Leptin receptor
*Lyn*: LYN proto-oncogene, Src family tyrosine kinase
*Msmo1*: Methylsterol monooxygenase 1
*Npas2*: Neuronal PAS domain protein 2
*Pde3b*: Phosphodiesterase 3B, cGMP-inhibited
*Pdk1*: Pyruvate dehydrogenase kinase, isozyme 1
*Per*: Period circadian clock
*Pik3r1*: Phosphoinositide-3-kinase
*Ppargc1b/Pgc1b*: Peroxisome proliferator-activated receptor gamma coactivator 1 beta
*Ppp1r1a*: Protein phosphatase 1, regulatory (inhibitor) subunit 1A
*Prf1*: Perforin 1 (pore-forming protein)
*Prkaa*: Protein kinase, AMP-activated, alpha
*Scd1*: Stearoyl-coenzyme A desaturase 1
*Sds*: Serine dehydratase
*Shc1*: SHC (Src homology 2 domain containing) transforming protein 1
*Srebf1*: Sterol regulatory element-binding transcription factor 1
*Sqle*: Squalene epoxidase
*Sqrdl*: Sulfide quinone reductase-like
