# Supplementary material for: Transcriptomic responses of the liver and adipose tissues to altered carbohydrate-fat ratio in diet: an isoenergetic study in young rats
Source: Genes Nutr. 2017 Apr 8;12:10. doi: 10.1186/s12263-017-0558-2 (PMC5385083; doi:10.1186/s12263-017-0558-2)
Supplement: Supplementary file 1 — Composition of diets. (DOCX 17 kb) [file 12263_2017_558_MOESM1_ESM.docx]

Online Resource1. Composition of diets

| Macronutrient | Ingredients | Low-fat diet | | | Middle-fat diet | | | High-fat diet | | |
| --- | --- | --- | --- | --- | --- | --- | --- | --- | --- | --- |
|  |  | Weight (g) | Energy (kcal) | Energy (%) | Weight (g) | Energy (kcal) | Energy (%) | Weight (g) | Energy (kcal) | Energy (%) |
| Protein  Amino acid | casein | 200 | 800 | 20 | 200 | 800 | 20 | 200 | 800 | 20 |
|  | L-cystine | 3 | 12 |  | 3 | 12 |  | 3 | 12 |  |
| Carbohydrate | corn starch | 448.2 | 1793 | 64 | 396 | 1584 | 59 | 144 | 576 | 34 |
|  | maltodextrin 10 | 100 | 400 |  | 100 | 400 |  | 100 | 400 |  |
|  | sucrose | 100 | 400 |  | 100 | 400 |  | 100 | 400 |  |
|  | cellulose BW200 | 50 | 0 | - | 50 | 0 | - | 50 | 0 | - |
| Fat | soybean oil | 17.8 | 160 | 15 | 41 | 369 | 20 | 153 | 1377 | 45 |
|  | lard | 50 | 450 |  | 50 | 450 |  | 50 | 450 |  |
|  | mineral mix S10026 | 10 | 0 | - | 10 | 0 | - | 10 | 0 | - |
|  | dicalcium phosphate | 13 | 0 | - | 13 | 0 | - | 13 | 0 | - |
|  | calcium carbonate | 5.5 | 0 | - | 5.5 | 0 | - | 5.5 | 0 | - |
|  | potassium citrate- 1H_2_O | 16.5 | 0 | - | 16.5 | 0 | - | 16.5 | 0 | - |
|  | vitamin mix V10001 | 10 | 40 | 1 | 10 | 40 | 1 | 10 | 40 | 1 |
|  | choline bitartrate | 2 | 0 | - | 2 | 0 | - | 2 | 0 | - |
|  | Total | 1026 | 4055 | 100 | 997 | 4055 | 100 | 857 | 4055 | 100 |
