# Supplementary figures and images for: Transcriptomic responses of the liver and adipose tissues to altered carbohydrate-fat ratio in diet: an isoenergetic study in young rats
Source: Genes Nutr. 2017 Apr 8;12:10. doi: 10.1186/s12263-017-0558-2 (PMC5385083; doi:10.1186/s12263-017-0558-2)

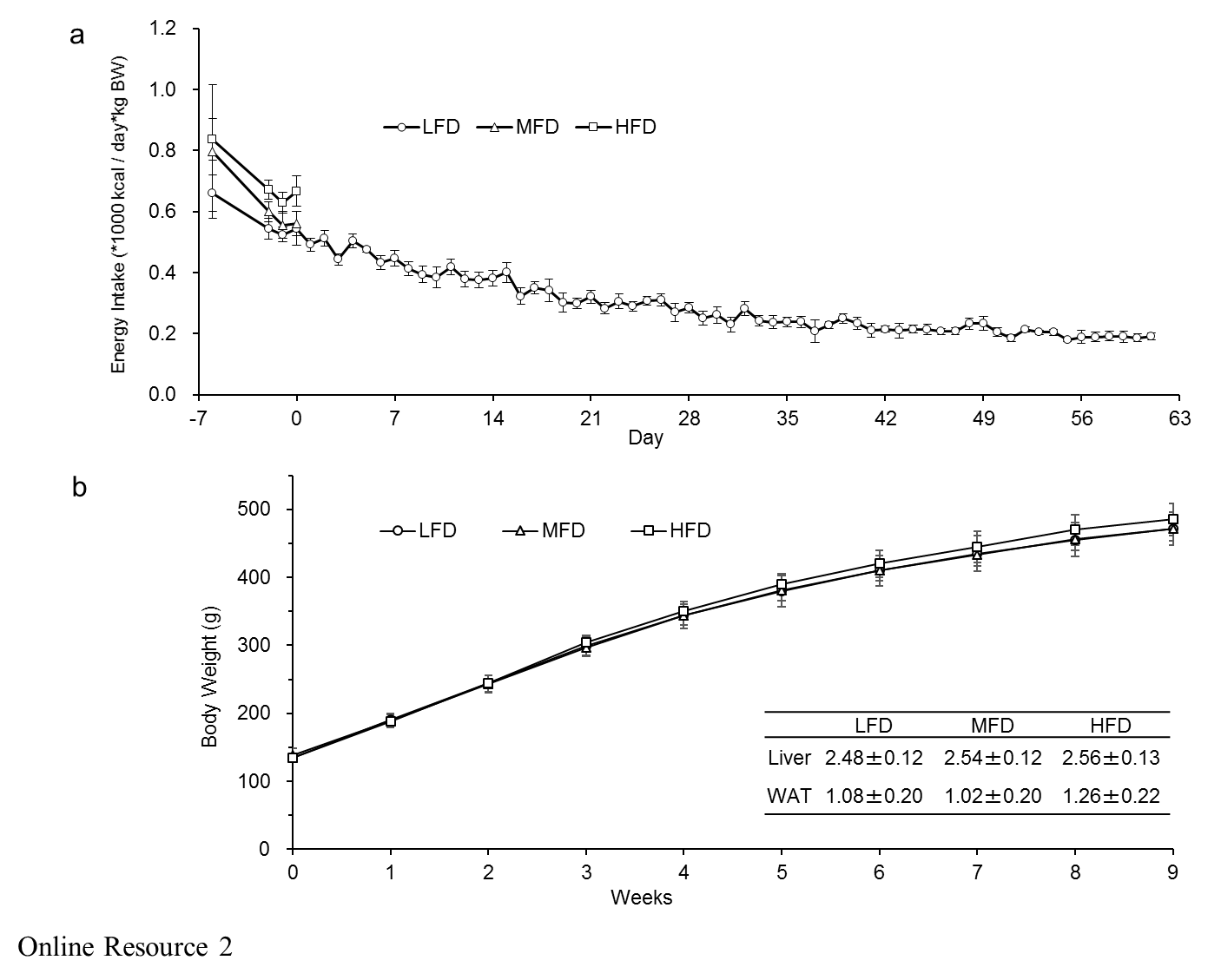

Supplement: Supplementary file 2 — Physical parameters of the animals. a, Energy intake during the experimental period. The intakes of the rats in the M- and H-groups were restricted to the average intake of the rats in the L-group. Data for the M- and H-groups after day 0 were omitted. b, Body and tissue weights. The inset represents the relative tissue weights (percent to body weight) at the time of sacrifice (week 9). Values are represented as means ± SD (n = 4–5). (DOCX 89 kb) [file 12263_2017_558_MOESM2_ESM.docx]
