# Supplementary material for: Transcriptomic responses of the liver and adipose tissues to altered carbohydrate-fat ratio in diet: an isoenergetic study in young rats
Source: Genes Nutr. 2017 Apr 8;12:10. doi: 10.1186/s12263-017-0558-2 (PMC5385083; doi:10.1186/s12263-017-0558-2)
Supplement: Supplementary file 3 — The list of liver LH186 + 189 genes that belongs to the GO terms located at the lowest level of hierarchy. (DOC 143 kb) [file 12263_2017_558_MOESM3_ESM.doc]

Online Resource 3. The list of Liver LH186+189 genes that belong to the GO terms located at the lowest level of hierarchy

| Probe ID | Gene Symbol | Description | L < H | L > H | GO:0019216 regulation of lipid metabolic process | GO:0006633 fatty acid biosynthetic process | GO:0008203 cholesterol metabolic process | GO:0033189 response to vitamin A | GO:0007623 circadian rhythm | GO:0045944 positive and GO:0000122 negative regulation of transcription from RNA polymerase II promoter | GO:0007568 aging | GO:0016525 negative regulation of angiogenesis | GO:0006882 cellular zinc ion homeostasis |
| --- | --- | --- | --- | --- | --- | --- | --- | --- | --- | --- | --- | --- | --- |
| 1379854_at | Abhd5 | abhydrolase domain containing 5 |  | L > H | + |  |  |  |  |  |  |  |  |
| 1389179_at | Cidea | cell death-inducing DFFA-like effector a | L < H |  | + |  |  |  |  |  |  |  |  |
| 1368592_at | Il1a | interleukin 1 alpha |  | L > H | + |  |  |  |  |  |  |  |  |
| 1368222_at | Nr3c1 | nuclear receptor subfamily 3, Glucocorticoid receptor |  | L > H | + |  |  |  |  |  |  |  |  |
| 1381886_at | Yy1 | YY1 transcription factor |  | L > H | + |  |  |  |  |  |  |  |  |
| 1386945_a_at | Prkab1 | Protein Kinase, AMP-Activated, Beta 1 Non-Catalytic Subunit |  | L > H | + | + |  |  |  |  |  |  |  |
| 1369654_at | Prkaa2 | Protein Kinase, AMP-Activated, Alpha 2 Catalytic Subunit |  | L > H | + | + | + |  |  |  |  |  |  |
| 1368520_at | Apoa4 | apolipoprotein A-IV |  | L > H | + |  | + |  |  |  |  |  |  |
| 1388426_at | Srebf1 | sterol regulatory element binding transcription factor 1 | L < H |  | + |  | + | + |  | + | + |  |  |
| 1367857_at | Fads1 | fatty acid desaturase 1 | L < H |  |  | + |  | + |  |  | + |  |  |
| 1368275_at | Msmo1 | methylsterol monooxygenase 1 | L < H |  |  | + |  |  |  |  |  |  |  |
| 1368934_at | Cyp4a1 | cytochrome P450 |  | L > H |  | + |  |  |  |  |  |  |  |
| 1387630_at | Elovl5 | ELOVL fatty acid elongase 5 |  | L > H |  | + |  |  |  |  |  |  |  |
| 1370355_at | Scd1 | stearoyl-Coenzyme A desaturase 1 |  | L > H |  | + |  |  |  |  |  |  |  |
| 1388188_at | Cyp7b1 | cytochrome P450 | L < H |  |  |  | + |  |  |  |  |  |  |
| 1368878_at 1388872_at | Idi1 | isopentenyl-diphosphate delta isomerase 1 | L < H |  |  |  | + |  |  |  |  |  |  |
| 1387017_at | Sqle | squalene epoxidase | L < H |  |  |  | + |  |  |  |  |  |  |
| 1370604_at 1370605_s_at 1371223_a_at | Lepr | leptin receptor |  | L > H |  |  | + |  |  |  |  |  |  |
| 1385640_at | Pcsk9 | proprotein convertase subtilisin/kexin type 9 | L < H |  |  |  | + |  |  |  |  |  |  |
| 1367648_at | Igfbp2 | insulin-like growth factor binding protein 2 | L < H |  |  |  |  | + |  |  | + |  |  |
| 1371143_at | Serpina7 | serpin peptidase inhibitor | L < H |  |  |  |  | + |  |  | + |  |  |
| 1368147_at | Dusp1 | dual specificity phosphatase 1 |  | L > H |  |  |  | + |  |  |  |  |  |
| 1370510_a_at | Arntl/Clock | aryl hydrocarbon receptor nuclear translocator-like |  | L > H |  |  |  |  | + | + |  |  |  |
| 1383439_at | Npas2/Clock | neuronal PAS domain protein 2 |  | L > H |  |  |  |  | + | + |  |  |  |
| 1370830_at | Egfr | epidermal growth factor receptor |  | L > H |  |  |  |  | + |  |  |  |  |
| 1370096_at | Prf1 | perforin 1 (pore forming protein) | L < H |  |  |  |  |  | + |  |  |  |  |
| 1368303_at | Per2 | period circadian clock 2 | L < H |  |  |  |  |  | + |  |  |  |  |
| 1378745_at | Per3 | period circadian clock 3 | L < H |  |  |  |  |  | + |  |  |  |  |
| 1387270_at | Hhex | hematopoietically expressed homeobox |  | L > H |  |  |  |  |  | + |  | + |  |
| 1379409_at | Atxn7 | ataxin 7 |  | L > H |  |  |  |  |  | + |  |  |  |
| 1382848_at | Foxa1 | forkhead box A1 |  | L > H |  |  |  |  |  | + |  |  |  |
| 1368711_at | Foxa2 | forkhead box A2 |  | L > H |  |  |  |  |  | + |  |  |  |
| 1396820_at | Hdac1 | histone deacetylase 1 |  | L > H |  |  |  |  |  | + |  |  |  |
| 1369012_at | Inhba | inhibin beta-A |  | L > H |  |  |  |  |  | + |  |  |  |
| 1368308_at | Myc | myelocytomatosis oncogene |  | L > H |  |  |  |  |  | + |  |  |  |
| 1371034_at 1387760_a_at | Onecut1 | one cut homeobox 1 |  | L > H |  |  |  |  |  | + |  |  |  |
| 1392715_at | Ppargc1b | peroxisome proliferator-activated receptor gamma coactivator 1 beta | L < H |  |  |  |  |  |  | + |  |  |  |
| 1378447_at | Thrap1 | Thyroid Hormone Receptor Associated Protein 1 |  | L > H |  |  |  |  |  | + |  |  |  |
| 1393809_at | Traf6 | TNF receptor-associated factor 6 |  | L > H |  |  |  |  |  | + |  |  |  |
| 1379469_at 1393491_at | Tbl1x | transducin (beta)-like 1 X-linked |  | L > H |  |  |  |  |  | + |  |  |  |
| 1387947_at 1382108_at | Mafb | v-maf avian musculoaponeurotic fibrosarcoma oncogene homolog B |  | L > H |  |  |  |  |  | + |  |  |  |
| 1372727_at 1369577_at | Socs2 | suppressor of cytokine signaling 2 |  | L > H |  |  |  |  |  | + |  |  |  |
| 1374529_at | Thbs1 | Thrombospondin 1 | L < H |  |  |  |  |  |  |  | + | + |  |
| 1369983_at | Ccl5 | chemokine (C-C motif) ligand 5 | L < H |  |  |  |  |  |  |  | + |  |  |
| 1370956_at | Dcn | decorin |  | L > H |  |  |  |  |  |  | + |  |  |
| 1371776_at 1370114_at | Pik3r1 | phosphoinositide-3-kinase |  | L > H |  |  |  |  |  |  | + |  |  |
| 1369693_a_at | Slc1a2 | solute carrier family 1 (glial high affinity glutamate transporter) | L < H |  |  |  |  |  |  |  | + |  |  |
| 1368877_at | Zfp354a | zinc finger protein 354A | L < H |  |  |  |  |  |  |  | + |  |  |
| 1387969_at | Cxcl10 | chemokine (C-X-C motif) ligand 10 | L < H |  |  |  |  |  |  |  |  | + |  |
| 1371250_at | Pf4 | platelet factor 4 | L < H |  |  |  |  |  |  |  |  | + |  |
| 1371237_a_at | LOC100362769, Mt1a, Ttr | hypothetical protein LOC100362769 , metallothionein 1a, transthyretin | L < H |  |  |  |  |  |  |  |  |  | + |
| 1388271_at | Mt2A | metallothionein 2A | L < H |  |  |  |  |  |  |  |  |  | + |
| 1374366_at | Slc39a4 | solute carrier family 39 (zinc transporter) |  | L > H |  |  |  |  |  |  |  |  | + |

Shaded cell entries: metabolic enzyme genes related to lipid.
