# Supplementary material for: Transcriptomic responses of the liver and adipose tissues to altered carbohydrate-fat ratio in diet: an isoenergetic study in young rats
Source: Genes Nutr. 2017 Apr 8;12:10. doi: 10.1186/s12263-017-0558-2 (PMC5385083; doi:10.1186/s12263-017-0558-2)
Supplement: Supplementary file 4 — The list of WAT LH235 + 336 genes that belong to the GO terms located at the lowest level of hierarchy. (DOC 190 kb) [file 12263_2017_558_MOESM4_ESM.doc]

Online Resource 4. The list of WAT LH235+336 genes that belong to the GO terms located at the lowest level of hierarchy

| Probe ID | Gene Symbol | Description | L < H | L > H | GO:0008610 lipid biosynthetic process | GO:0006635 fatty acid beta-oxidation | GO:0045444 fat cell differentiation | GO:0006006 glucose metabolic process | GO:0032868 response to insulin stimulus | GO:0007584 response to nutrient | GO:0060348 bone development | GO:0001503 ossification |
| --- | --- | --- | --- | --- | --- | --- | --- | --- | --- | --- | --- | --- |
| 1367660_at | Fabp3 | fatty acid binding protein 3, muscle and heart | L < H |  | + | + |  |  | + |  |  |  |
| 1370355_at | Scd1 | stearoyl-Coenzyme A desaturase 1 |  | L > H | + |  | + |  |  |  |  |  |
| 1386917_at | Pc | pyruvate carboxylase |  | L > H | + |  |  | + |  |  |  |  |
| 1387174_a_at | Star | steroidogenic acute regulatory protein |  | L > H | + |  |  |  | + | + |  |  |
| 1367894_at | Insig1 | insulin induced gene 1 |  | L > H | + |  |  |  | + |  | + |  |
| 1368527_at | Ptgs2 | prostaglandin-endoperoxide synthase 2 |  | L > H | + |  |  |  |  | + | + | + |
| 1368003_at | Aldh1a2 | aldehyde dehydrogenase 1 family, member A2 |  | L > H | + |  |  |  |  | + |  |  |
| 1372318_at,  1394401_at | [ELOVL6](http://betacellgenebank.ulb.ac.be/page/GeneMore/display/seq_id/BE116152) | ELOVL family member 6 |  | L > H | + |  |  |  |  |  |  |  |
| 1391544_at | Pnpla3 | patatin-like phospholipase domain containing 3 |  | L > H | + |  |  |  |  |  |  |  |
| 1380665_at | Abhd5 | abhydrolase domain containing 5 |  | L > H | + |  |  |  |  |  |  |  |
| 1387538_at | Acaca | acetyl-CoA carboxylase alpha |  | L > H | + |  |  |  |  |  |  |  |
| 1367854_at | Acly | ATP citrate lyase |  | L > H | + |  |  |  |  |  |  |  |
| 1375944_at | Acss2 | acyl-CoA synthetase short-chain family member 2 |  | L > H | + |  |  |  |  |  |  |  |
| 1381574_at | Agmo | alkylglycerol monooxygenase |  | L > H | + |  |  |  |  |  |  |  |
| 1368692_a_at | Chka | choline kinase alpha |  | L > H | + |  |  |  |  |  |  |  |
| 1370235_at | Dbi | diazepam binding inhibitor (GABA receptor modulator, acyl-CoA binding protein) |  | L > H | + |  |  |  |  |  |  |  |
| 1367915_at | Dgat1 | diacylglycerol O-acyltransferase 1 |  | L > H | + |  |  |  |  |  |  |  |
| 1371710_at | Etnk1 | ethanolamine kinase 1 |  | L > H | + |  |  |  |  |  |  |  |
| 1367707_at | Fasn | fatty acid synthase |  | L > H | + |  |  |  |  |  |  |  |
| 1396208_at | Ggt5 | gamma-glutamyltransferase 5 | L < H |  | + |  |  |  |  |  |  |  |
| 1367932_at | Hmgcs1 | 3-hydroxy-3-methylglutaryl-CoA synthase 1 (soluble) |  | L > H | + |  |  |  |  |  |  |  |
| 1368051_at | Hsd17b12 | hydroxysteroid (17-beta) dehydrogenase 12 |  | L > H | + |  |  |  |  |  |  |  |
| 1376813_at | Lpcat3 | lysophosphatidylcholine acyltransferase 3 |  | L > H | + |  |  |  |  |  |  |  |
| 1391264_at | Lrrn4 | leucine rich repeat neuronal 4 |  | L > H | + |  |  |  |  |  |  |  |
| 1380013_at | Pnpla3 | patatin-like phospholipase domain containing 3 |  | L > H | + |  |  |  |  |  |  |  |
| 1386956_at | Scarb1 | scavenger receptor class B, member 1 | L < H |  | + |  |  |  |  |  |  |  |
| 1381997_at | Adipoq | adiponectin, C1Q and collagen domain containing |  | L > H |  | + | + | + |  | + |  |  |
| 1367829_at | Echs1, LOC100911186 | enoyl CoA hydratase, short chain, 1, mitochondrial, enoyl-CoA hydratase, mitochondrial-like |  | L > H |  | + |  |  |  |  |  |  |
| 1367659_s_at | Eci1 | enoyl-CoA delta isomerase 1 |  | L > H |  | + |  |  |  |  |  |  |
| 1388908_at | Eci2 | enoyl-CoA delta isomerase 2 |  | L > H |  | + |  |  |  |  |  |  |
| 1368283_at | Ehhadh | enoyl-CoA, hydratase/3-hydroxyacyl CoA dehydrogenase |  | L > H |  | + |  |  |  |  |  |  |
| 1371519_at | Etfdh | electron-transferring-flavoprotein dehydrogenase |  | L > H |  | + |  |  |  |  |  |  |
| 1371775_at | LOC100912409 | short/branched chain specific acyl-CoA dehydrogenase, mitochondrial-like | L < H |  |  | + |  |  |  |  |  |  |
| 1383836_at | RETN | resistin |  | L > H |  |  | + |  | + |  |  |  |
| 1368126_at | Aacs | acetoacetyl-CoA synthetase |  | L > H |  |  | + |  |  | + |  |  |
| 1379815_at | Tcf7l2 | Transcription factor 7-like 2, T-cell specific, HMG-box |  | L > H |  |  | + |  |  |  |  |  |
| 1383848_at | ADRB1 | adrenergic, beta-1-, receptor |  | L > H |  |  | + |  |  |  |  |  |
| 1368780_at | Adrb3 | adrenoceptor beta 3 |  | L > H |  |  | + |  |  |  |  |  |
| 1374626_at | Lrg1 | leucine-rich alpha-2-glycoprotein 1 | L < H |  |  |  | + |  |  |  |  |  |
| 1387074_at | Rgs2 | regulator of G-protein signaling 2 |  | L > H |  |  | + |  |  |  |  |  |
| 1393260_at | Wfdc21 | WAP four-disulfide core domain 21 |  | L > H |  |  | + |  |  |  |  |  |
| 1371776_at | PIK3R1 | phosphoinositide-3-kinase, regulatory subunit 1 (alpha) |  | L > H |  |  |  | + | + |  |  |  |
| 1387748_at | Lep | leptin | L < H |  |  |  |  | + | + |  |  |  |
| 1373140_at | Il6st | interleukin 6 signal transducer | L < H |  |  |  |  | + |  | + |  |  |
| 1367571_a_at | Igf2 | insulin-like growth factor 2 |  | L > H |  |  |  | + |  |  | + | + |
| 1378960_at, 1371363_at | GPD1 | Glycerol-3-phosphate dehydrogenase 1 (soluble) |  | L > H |  |  |  | + |  |  |  |  |
| 1369268_at | Atf3 | activating transcription factor 3 |  | L > H |  |  |  | + |  |  |  |  |
| 1378925_at | Crem | cAMP responsive element modulator |  | L > H |  |  |  | + |  |  |  |  |
| 1370026_at | Cryab | crystallin, alpha B | L < H |  |  |  |  | + |  |  |  |  |
| 1383698_at | Pdha1 | pyruvate dehydrogenase (lipoamide) alpha 1 |  | L > H |  |  |  | + |  |  |  |  |
| 1368079_at | Pdk1 | pyruvate dehydrogenase kinase, isozyme 1 |  | L > H |  |  |  | + |  |  |  |  |
| 1386968_at | Ppp1r1a | protein phosphatase 1, regulatory (inhibitor) subunit 1A |  | L > H |  |  |  | + |  |  |  |  |
| 1386900_at | Serp1 | stress-associated endoplasmic reticulum protein 1 |  | L > H |  |  |  | + |  |  |  |  |
| 1379065_at | Serpina12 | serpin peptidase inhibitor, clade A (alpha-1 antiproteinase, antitrypsin), member 12 |  | L > H |  |  |  | + |  |  |  |  |
| 1369940_at | Taldo1 | transaldolase 1 |  | L > H |  |  |  | + |  |  |  |  |
| 1382346_at | LYN | LYN proto-oncogene, Src family tyrosine kinase | L < H |  |  |  |  |  | + |  |  |  |
| 1396965_at | [FOXO1](http://www.uniprot.org/uniprot/Q12778) | Forkhead box O1A |  | L > H |  |  |  |  | + |  |  |  |
| 1379274_at | --- | --- |  | L > H |  |  |  |  | + |  |  |  |
| 1370534_at | Acvr1c | activin A receptor, type IC |  | L > H |  |  |  |  | + |  |  |  |
| 1368270_at | Apobec1 | apolipoprotein B mRNA editing enzyme, catalytic polypeptide 1 | L < H |  |  |  |  |  | + |  |  |  |
| 1371076_at | Cyp2b1, Cyp2b2, LOC100909962 | cytochrome P450, family 2, subfamily b, polypeptide 1, cytochrome P450, family 2, subfamily b, polypeptide 2, cytochrome P450 2B1-like | L < H |  |  |  |  |  | + |  |  |  |
| 1370047_at | Enpp1 | ectonucleotide pyrophosphatase/phosphodiesterase 1 |  | L > H |  |  |  |  | + |  |  |  |
| 1369157_at | Pde3b | phosphodiesterase 3B, cGMP-inhibited |  | L > H |  |  |  |  | + |  |  |  |
| 1377594_at | Shc1 | SHC (Src homology 2 domain containing) transforming protein 1 |  | L > H |  |  |  |  | + |  |  |  |
| 1388153_at | Acsl1 | acyl-CoA synthetase long-chain family member 1 |  | L > H |  |  |  |  |  | + |  |  |
| 1370906_at | Bckdhb | branched chain keto acid dehydrogenase E1, beta polypeptide |  | L > H |  |  |  |  |  | + |  |  |
| 1387037_at | Cubn | cubilin (intrinsic factor-cobalamin receptor) | L < H |  |  |  |  |  |  | + |  |  |
| 1368147_at | Dusp1 | dual specificity phosphatase 1 |  | L > H |  |  |  |  |  | + |  |  |
| 1367627_at | Gatm | glycine amidinotransferase (L-arginine:glycine amidinotransferase) | L < H |  |  |  |  |  |  | + |  |  |
| 1386872_at | Igf2r | insulin-like growth factor 2 receptor |  | L > H |  |  |  |  |  | + |  |  |
| 1367648_at | Igfbp2 | insulin-like growth factor binding protein 2 |  | L > H |  |  |  |  |  | + |  |  |
| 1388569_at | Serpinf1 | serpin peptidase inhibitor, clade F (alpha-2 antiplasmin, pigment epithelium derived factor), member 1 | L < H |  |  |  |  |  |  | + |  |  |
| 1368989_at | Timp3 | TIMP metallopeptidase inhibitor 3 | L < H |  |  |  |  |  |  | + |  |  |
| 1387232_at | Bmp4 | bone morphogenetic protein 4 | L < H |  |  |  |  |  |  | + | + | + |
| 1367563_at | Sparc | secreted protein, acidic, cysteine-rich (osteonectin) | L < H |  |  |  |  |  |  | + | + | + |
| 1367581_a_at | Spp1 | secreted phosphoprotein 1 | L < H |  |  |  |  |  |  | + | + | + |
| 1369773_at | Bmp3 | bone morphogenetic protein 3 |  | L > H |  |  |  |  |  |  | + | + |
| 1393579_at | Chrdl1 | chordin-like 1 | L < H |  |  |  |  |  |  |  | + | + |
| 1371951_at | Fhl2 | four and a half LIM domains 2 | L < H |  |  |  |  |  |  |  | + | + |
| 1388039_a_at | Gabbr1 | gamma-aminobutyric acid (GABA) B receptor 1 |  | L > H |  |  |  |  |  |  | + | + |
| 1368187_at | Gpnmb | glycoprotein (transmembrane) nmb | L < H |  |  |  |  |  |  |  | + | + |
| 1373410_at | Mef2c | myocyte enhancer factor 2C | L < H |  |  |  |  |  |  |  | + | + |
| 1369968_at | Ptn | pleiotrophin | L < H |  |  |  |  |  |  |  | + | + |
| 1367859_at | Tgfb3 | transforming growth factor, beta 3 | L < H |  |  |  |  |  |  |  | + | + |
| 1376047_at | Papss2 | 3'-phosphoadenosine 5'-phosphosulfate synthase 2 | L < H |  |  |  |  |  |  |  | + |  |

Shaded cell entries: metabolic enzyme genes related to lipid.
