# Supplementary material for: Transcriptomic responses of the liver and adipose tissues to altered carbohydrate-fat ratio in diet: an isoenergetic study in young rats
Source: Genes Nutr. 2017 Apr 8;12:10. doi: 10.1186/s12263-017-0558-2 (PMC5385083; doi:10.1186/s12263-017-0558-2)
Supplement: Supplementary file 5 — The list of BAT LH212 + 405 genes that belong to the GO terms located at the lowest level of hierarchy. (DOC 213 kb) [file 12263_2017_558_MOESM5_ESM.doc]

Online Resource 5. The list of BAT LH212+405 genes that belong to the GO terms located at the lowest level of hierarchy

| Probe ID | Gene Symbol | Description | L < H | L > H | GO:0006631 fatty acid metabolic process | GO:0006695 cholesterol biosynthetic process | GO:0055114 oxidation reduction | GO:0010033 response to organic substance | GO:0006936 muscle contraction |
| --- | --- | --- | --- | --- | --- | --- | --- | --- | --- |
| 1367857_at | Fads1 | fatty acid desaturase 1 |  | L > H | + |  | + | + |  |
| 1381574_at | Agmo | alkylglycerol monooxygenase |  | L > H | + |  | + |  |  |
| 1368283_at | Ehhadh | enoyl-CoA, hydratase/3-hydroxyacyl CoA dehydrogenase |  | L > H | + |  | + |  |  |
| 1367708_a_at | Fasn | fatty acid synthase |  | L > H | + |  | + |  |  |
| 1394550_at | Faxdc2 | fatty acid hydroxylase domain containing 2 |  | L > H | + |  | + |  |  |
| 1367668_a_at | LOC100912469, Scd | acyl-CoA desaturase 2-like, stearoyl-CoA desaturase (delta-9-desaturase) |  | L > H | + |  | + |  |  |
| 1390777_at | Sc5d | sterol-C5-desaturase |  | L > H | + |  | + |  |  |
| 1370355_at | Scd1 | stearoyl-Coenzyme A desaturase 1 |  | L > H | + |  | + |  |  |
| 1398170_at | --- | --- |  | L > H | + |  |  | + |  |
| 1387538_at | Acaca | acetyl-CoA carboxylase alpha |  | L > H | + |  |  | + |  |
| 1373778_at | ACC2 | acetyl-CoA carboxylase beta |  | L > H | + |  |  | + |  |
| 1387101_at | Acsl4 | acyl-CoA synthetase long-chain family member 4 |  | L > H | + |  |  | + |  |
| 1386926_at | Acsl5 | acyl-CoA synthetase long-chain family member 5 |  | L > H | + |  |  | + |  |
| 1376522_at | FABP3 | Fatty acid binding protein 3 | L < H |  | + |  |  | + |  |
| 1387748_at | Lep | leptin |  | L > H | + |  |  | + |  |
| 1387278_at | Ppara | peroxisome proliferator activated receptor alpha | L < H |  | + |  |  | + |  |
| 1367789_at | Slc27a1 | solute carrier family 27 (fatty acid transporter), member 1 |  | L > H | + |  |  | + |  |
| 1392952_at | Acsf2 | acyl-CoA synthetase family member 2 |  | L > H | + |  |  |  |  |
| 1377407_at | Acsm5 | acyl-CoA synthetase medium-chain family member 5 |  | L > H | + |  |  |  |  |
| 1394401_at, 1372318_at | Elovl6 | ELOVL fatty acid elongase 6 |  | L > H | + |  |  |  |  |
| 1368150_at | Slc27a2 | solute carrier family 27 (fatty acid transporter), member 2 | L < H |  | + |  |  |  |  |
| 1367894_at | Insig1 | insulin induced gene 1 |  | L > H |  | + |  | + |  |
| 1386990_at | Ebp | emopamil binding protein (sterol isomerase) |  | L > H |  | + |  |  |  |
| 1367667_at | Fdps | farnesyl diphosphate synthase |  | L > H |  | + |  |  |  |
| 1372973_at | Lss | lanosterol synthase (2,3-oxidosqualene-lanosterol cyclase) |  | L > H |  | + |  |  |  |
| 1373243_at | Pmvk | phosphomevalonate kinase |  | L > H |  | + |  |  |  |
| 1367856_at | G6pd | glucose-6-phosphate dehydrogenase |  | L > H |  | + | + | + |  |
| 1370808_at | Cyb5r3 | cytochrome b5 reductase 3 |  | L > H |  | + | + |  |  |
| 1367979_s_at | Cyp51 | cytochrome P450, family 51 |  | L > H |  | + | + |  |  |
| 1368189_at | Dhcr7 | 7-dehydrocholesterol reductase |  | L > H |  | + | + |  |  |
| 1389906_at | Fdft1 | farnesyl diphosphate farnesyl transferase 1 |  | L > H |  | + | + |  |  |
| 1392988_at | Nsdhl | NAD(P) dependent steroid dehydrogenase-like |  | L > H |  | + | + |  |  |
| 1368990_at | Cyp1b1 | cytochrome P450, family 1, subfamily b, polypeptide 1 |  | L > H |  |  | + | + |  |
| 1370080_at | Hmox1 | heme oxygenase (decycling) 1 |  | L > H |  |  | + | + |  |
| 1369954_at | Idh1 | isocitrate dehydrogenase 1 (NADP+), soluble |  | L > H |  |  | + | + |  |
| 1370870_at | Me1 | malic enzyme 1, NADP(+)-dependent, cytosolic |  | L > H |  |  | + | + |  |
| 1389548_at | Adhfe1 | alcohol dehydrogenase, iron containing, 1 | L < H |  |  |  | + |  |  |
| 1370902_at | Akr1b8 | aldo-keto reductase family 1, member B8 | L < H |  |  |  | + |  |  |
| 1370708_a_at | Akr1c14 | aldo-keto reductase family 1, member C14 |  | L > H |  |  | + |  |  |
| 1373085_at | Cbr3 | carbonyl reductase 3 |  | L > H |  |  | + |  |  |
| 1377666_at | Chdh | choline dehydrogenase |  | L > H |  |  | + |  |  |
| 1367739_at | Cox8b | cytochrome c oxidase, subunit VIIIb | L < H |  |  |  | + |  |  |
| 1371795_at | Cyb5b | cytochrome b5 type B (outer mitochondrial membrane) |  | L > H |  |  | + |  |  |
| 1367871_at | Cyp2e1 | cytochrome P450, family 2, subfamily e, polypeptide 1 | L < H |  |  |  | + |  |  |
| 1398710_at | Cyp2u1 | cytochrome P450, family 2, subfamily u, polypeptide 1 |  | L > H |  |  | + |  |  |
| 1370399_at | Cyp4b1 | cytochrome P450, family 4, subfamily b, polypeptide 1 |  | L > H |  |  | + |  |  |
| 1367909_at | Dcxr | dicarbonyl L-xylulose reductase | L < H |  |  |  | + |  |  |
| 1369279_at | Dhrs9 | dehydrogenase/reductase (SDR family) member 9 |  | L > H |  |  | + |  |  |
| 1390172_at | Dhtkd1 | dehydrogenase E1 and transketolase domain containing 1 | L < H |  |  |  | + |  |  |
| 1377635_at | Fmo2 | flavin containing monooxygenase 2 | L < H |  |  |  | + |  |  |
| 1388629_at | Impdh2 | IMP (inosine 5'-monophosphate) dehydrogenase 2 |  | L > H |  |  | + |  |  |
| 1368514_at | Maob | monoamine oxidase B |  | L > H |  |  | + |  |  |
| 1372790_at | Mdh1 | malate dehydrogenase 1, NAD (soluble) |  | L > H |  |  | + |  |  |
| 1389572_at | Me3 | malic enzyme 3, NADP(+)-dependent, mitochondrial | L < H |  |  |  | + |  |  |
| 1383698_at | Pdha1 | pyruvate dehydrogenase (lipoamide) alpha 1 |  | L > H |  |  | + |  |  |
| 1385393_at | PPARGC1A | PPARγ coactivator 1α | L < H |  |  |  | + |  |  |
| 1375862_at | Pxdn | peroxidasin homolog (Drosophila) |  | L > H |  |  | + |  |  |
| 1373128_at | Rcn3 | reticulocalbin 3, EF-hand calcium binding domain |  | L > H |  |  | + |  |  |
| 1371358_at | RGD1560015, Tecr | similar to glycoprotein, synaptic 2, trans-2,3-enoyl-CoA reductase |  | L > H |  |  | + |  |  |
| 1377213_at | ERN1 | endoplasmic reticulum to nucleus signaling 1 |  | L > H |  |  |  | + |  |
| 1392655_at | SREBP2 | [Sterol-regulatory element-binding protein-2](http://www.ncbi.nlm.nih.gov/pubmed/12119189) |  | L > H |  |  |  | + |  |
| 1368769_at | Abcb11 | ATP-binding cassette, subfamily B (MDR/TAP), member 11 |  | L > H |  |  |  | + |  |
| 1370009_at | Apoc3 | apolipoprotein C-III | L < H |  |  |  |  | + |  |
| 1367599_at | Atp5g1, LOC689961 | ATP synthase, H+ transporting, mitochondrial Fo complex, subunit C1 (subunit 9), similar to ATP synthase lipid-binding protein, mitochondrial precursor |  | L > H |  |  |  | + |  |
| 1386922_at | Car2 | carbonic anhydrase 2 | L < H |  |  |  |  | + |  |
| 1369983_at | Ccl5 | chemokine (C-C motif) ligand 5 | L < H |  |  |  |  | + |  |
| 1370810_at | Ccnd2 | cyclin D2 |  | L > H |  |  |  | + |  |
| 1368490_at | Cd14 | CD14 molecule |  | L > H |  |  |  | + |  |
| 1370864_at | Col1a1 | collagen, type I, alpha 1 |  | L > H |  |  |  | + |  |
| 1368290_at | Cyr61 | cysteine-rich, angiogenic inducer, 61 | L < H |  |  |  |  | + |  |
| 1368321_at | Egr1 | early growth response 1 |  | L > H |  |  |  | + |  |
| 1375043_at | FOS | FBJ murine osteosarcoma viral oncogene homolog | L < H |  |  |  |  | + |  |
| 1370522_at | Gcgr | glucagon receptor |  | L > H |  |  |  | + |  |
| 1387906_a_at | Gnas | GNAS complex locus | L < H |  |  |  |  | + |  |
| 1377993_at | Gng13 | guanine nucleotide binding protein (G protein), gamma 13 |  | L > H |  |  |  | + |  |
| 1387023_at | Gstm7 | glutathione S-transferase, mu 7 | L < H |  |  |  |  | + |  |
| 1368247_at | Hspa1a, Hspa1b | heat shock 70kD protein 1A, heat shock 70kD protein 1B (mapped) |  | L > H |  |  |  | + |  |
| 1378002_at | Hspa4l | heat shock protein 4-like |  | L > H |  |  |  | + |  |
| 1387028_a_at | Id1 | inhibitor of DNA binding 1 | L < H |  |  |  |  | + |  |
| 1368870_at | Id2 | inhibitor of DNA binding 2 |  | L > H |  |  |  | + |  |
| 1370333_a_at | Igf1 | insulin-like growth factor 1 |  | L > H |  |  |  | + |  |
| 1367648_at | Igfbp2 | insulin-like growth factor binding protein 2 | L < H |  |  |  |  | + |  |
| 1376089_at | LDLR | low density lipoprotein receptor |  | L > H |  |  |  | + |  |
| 1381363_at | LRP6 | low density lipoprotein receptor-related protein 6 | L < H |  |  |  |  | + |  |
| 1388300_at | Mgst3 | microsomal glutathione S-transferase 3 | L < H |  |  |  |  | + |  |
| 1370301_at | Mmp2 | matrix metallopeptidase 2 |  | L > H |  |  |  | + |  |
| 1368308_at | Myc | myelocytomatosis oncogene |  | L > H |  |  |  | + |  |
| 1368376_at | Nr0b2 | nuclear receptor subfamily 0, group B, member 2, small heterodimer partner (SHP) | L < H |  |  |  |  | + |  |
| 1369067_at | Nr4a3 | nuclear receptor subfamily 4, group A, member 3, orphan |  | L > H |  |  |  | + |  |
| 1385148_at | P2rx1 | purinergic receptor P2X, ligand-gated ion channel, 1 |  | L > H |  |  |  | + |  |
| 1371776_at | PIK3R1 | phosphoinositide-3-kinase, regulatory subunit 1 (alpha) |  | L > H |  |  |  | + |  |
| 1370114_a_at | Pik3r1 | phosphoinositide-3-kinase, regulatory subunit 1 (alpha) |  | L > H |  |  |  | + |  |
| 1390383_at | Plin2 | perilipin 2 |  | L > H |  |  |  | + |  |
| 1392715_at | Ppargc1b | peroxisome proliferator-activated receptor gamma, coactivator 1 beta | L < H |  |  |  |  | + |  |
| 1376938_at | PPP2R2A | Protein phosphatase 2 (formerly 2A), regulatory subunit B (PR 52), alpha isoform | L < H |  |  |  |  | + |  |
| 1382189_at | Sdc2 | syndecan 2 |  | L > H |  |  |  | + |  |
| 1388569_at | Serpinf1 | serpin peptidase inhibitor, clade F (alpha-2 antiplasmin, pigment epithelium derived factor), member 1 |  | L > H |  |  |  | + |  |
| 1367802_at | Sgk1 | serum/glucocorticoid regulated kinase 1 | L < H |  |  |  |  | + |  |
| 1375266_at | Slc6a9 | Solute carrier family 6 (neurotransmitter transporter, glycine), member 9 |  | L > H |  |  |  | + |  |
| 1372727_at | Socs2 | Suppressor of cytokine signaling 2 |  | L > H |  |  |  | + |  |
| 1371113_a_at | Tfrc | transferrin receptor | L < H |  |  |  |  | + |  |
| 1377632_at | Timp4 | tissue inhibitor of metalloproteinase 4 | L < H |  |  |  |  | + |  |
| 1371019_at | Trib1 | tribbles pseudokinase 1 | L < H |  |  |  |  | + |  |
| 1371131_a_at | Txnip | thioredoxin interacting protein |  | L > H |  |  |  | + |  |
| 1371249_at | Xbp1 | X-box binding protein 1 |  | L > H |  |  |  | + |  |
| 1369928_at | Acta1 | actin, alpha 1, skeletal muscle | L < H |  |  |  |  | + |  |
| 1387768_at | Mb | myoglobin | L < H |  |  |  |  | + | + |
| 1381296_at | --- | --- |  | L > H |  |  |  |  | + |
| 1367962_at | Actn3 | actinin alpha 3 | L < H |  |  |  |  |  | + |
| 1387811_at | Agt | angiotensinogen (serpin peptidase inhibitor, clade A, member 8) | L < H |  |  |  |  |  | + |
| 1376968_at | Mybpc2 | myosin binding protein C, fast-type | L < H |  |  |  |  |  | + |
| 1370971_at | Myh1 | myosin, heavy chain 1, skeletal muscle, adult, myosin, heavy chain 2, skeletal muscle, adult, myosin, heavy chain 8, skeletal muscle, perinatal | L < H |  |  |  |  |  | + |
| 1370896_a_at | Myh11 | myosin, heavy chain 11, smooth muscle |  | L > H |  |  |  |  | + |
| 1370033_at | Myl1 | myosin, light chain 1 | L < H |  |  |  |  |  | + |
| 1387787_at | Mylpf | myosin light chain, phosphorylatable, fast skeletal muscle | L < H |  |  |  |  |  | + |
| 1372195_at | Tnnc2 | troponin C type 2 (fast) | L < H |  |  |  |  |  | + |
| 1367964_at | Tnni2 | troponin I type 2 (skeletal, fast) | L < H |  |  |  |  |  | + |
| 1371247_at | Tnnt3 | troponin T type 3 (skeletal, fast) | L < H |  |  |  |  |  | + |
| 1371241_x_at | Tpm1 | tropomyosin 1, alpha | L < H |  |  |  |  |  | + |
| 1371241_x_at | Tpm1 | tropomyosin 1, alpha | L < H |  |  |  |  |  | + |

Shaded cell entries: metabolic enzyme genes related to lipid.
